# DNA Binding and Antitumor Activity of *α*-Diimineplatinum(II) and Palladium(II) Dithiocarbamate Complexes

**DOI:** 10.1155/2011/394506

**Published:** 2011-10-30

**Authors:** Hassan Mansouri-Torshizi, Maryam Saeidifar, Fatemeh Khosravi, Adeleh Divsalar, Ali Akbar Saboury, Fatemeh Hassani

**Affiliations:** ^1^Department of Chemistry, University of Sistan and Baluchestan, Zahedan 98167-45345, Iran; ^2^Nanotechnology and Advanced Materials Department, Materials and Energy Research Center, Karaj 31787-316, Iran; ^3^Department of Biological Sciences, Tarbiat Moallem University, Tehran 13145-1384, Iran; ^4^Institute of Biochemistry and Biophysics, University of Tehran, Tehran 13145-1384, Iran

## Abstract

The two water-soluble designed platinum(II) complex, [Pt(Oct-dtc)(bpy)]NO_3_ (Oct-dtc = Octyldithiocarbamate and bpy = 2,2′ -bipyridine) and palladium(II) complex, [Pd(Oct-dtc)(bpy)]NO_3_, have been synthesized and characterized by elemental analyses, molar conductivity measurements, IR, ^1^H NMR, and electronic spectra studies. Studies of antitumor activity of these complexes against human cell tumor lines (K562) have been carried out. They show Ic_50_ values lower than that of cisplatin. The complexes have been investigated for their interaction with calf thymus DNA (CT-DNA) by utilizing the electronic absorption spectroscopy, fluorescence spectra, and ethidium bromide displacement and gel filtration techniques. Both of these water-soluble complexes bound cooperatively and intercalatively to the CT-DNA at very low concentrations. Several binding and thermodynamic parameters are also described.

## 1. Introduction

In order to reduce the toxicity of cisplatin, that is, the well-known anticancer drug, and modulate its activity, a new strategy is the design of novel metal complexes containing N and S donor ligands [1–3]. This interest has probably initiated from detoxicant properties of sulfur-containing ligands against heavy metal intoxication [4]. As an example is the use of sodium diethyldithiocarbamate (DDTC) in the treatment of patients with acute poisoning of nickel carbonyl, arsenic, and thallium [5]. A further interest in this chelating ligand has arisen due to its highly selective use to protect a variety of animal species from renal, gastrointestinal, and bone marrow toxicity, induced by cisplatin without inhibition of cisplatin's antitumor effect [6–9]. In addition, diethyldithiocarbamate has remarkable property of reversing platinum binding to macromolecules responsible for host toxicity. However, it does not interfere with the tumoricidal Pt-DNA interaction in the tumor cell. Thus, the protective action of the dithiocarbamate against the toxicity of cisplatin seems to be the formation of its stable platinum dithiocarbamate complexes [7, 10].

A large number of analogs of cisplatin have been tested, and it has been reported that many active complexes could react with cell DNA and inhibit its synthesis [11–14]. Recently, significant attention has been focused on the DNA binding properties of dithiocarbamate metal complexes [15–17] and several dithiocarbamate derivatives have been investigated as potential biologically active agents [18–23]. Among which platinum and palladium complexes of dithiocarbamates have gained considerable interest due-to-their potential antitumor properties [24–29].

It has also known that dithiocarbamate complexes of [M(NN)(SS)] type, with diimine (NN) as a *π*-electron acceptor and dithiocarbamate (SS) as an electron donor, exhibit intramolecular mixed metal ligand to ligand charge transfer bands [30, 31]. This band appears in a region where CT-DNA has no absorption and thus has been widely used for the absorption spectrophotometric binding studies.

In this work, we have chosen a bioactive octyldithiocarbamate ligand whose structure not only resembles a great number of antibacterial, antiviral, anthelmintic, and insecticidal agent, but also can be used as inhibitor of cisplatin-induced nephrotoxicity [32] while as diimine we have employed 2,2′-bipyridine (bpy) whose structure adopts a planar conformation when it chelates to Pt(II) or Pd(II) center [33], and thus can intercalate in CT-DNA. These complexes have been tested against human cell tumor line K562. In order to confirm the mode of binding of these complexes to CT-DNA, detailed interaction studies of them with CT-DNA are attempted.

## 2. Experimental

### 2.1. Materials and Methods

Octylamine and carbon disulfide were purchased from Aldrich (England). Palladium(II) chloride was obtained from Fluka (Switzerland). Potassium tetrachloroplatinate, 2,2′-bipyridine, highly polymerized calf thymus DNA sodium salt, and Tris-HCl buffer were obtained from Merck (Germany). Other chemicals used were of analytical reagent or higher purity grade. [Pt(bpy)Cl_2_] and [Pd(bpy)Cl_2_] were prepared by the procedures described in the literature [34]. Solvents used were of reagent grade and purified before use by the standard methods. All the experiments involving interaction of the complexes with CT-DNA were performed in Tris-HCl buffer (20 mM) of pH = 7.0 medium containing 20 mM NaCl. Monitoring the ratio of the absorbance at 260 to that of 280 nm checks purity of CT-DNA. The solution gave a ratio of >1.8 at *A*_260_/*A*_280_, indicating that CT-DNA is sufficiently free from protein [35, 36]. The CT-DNA concentration per nucleotide was determined by absorption spectroscopy using the known molar extinction coefficient value of 6600 M^−1 ^cm-^1^ at 260 nm [37]. 

Electronic absorption spectra of the title metal complexes were measured on a J_AS.CO_ UV/Vis-7850 recording spectrophotometer. Infrared spectra of the metal complexes were recorded on a J_AS.CO_-460 Plus FT-IR spectrophotometer in the range of 4000–400 cm^−1^ in KBr pellets. Microchemical analysis of carbon, hydrogen, and nitrogen for the complexes were carried out on a Herause CHNO-RAPID elemental analyzer. ^1^H NMR spectra were recorded on a Brucker DRX-500 Avance spectrometer at 500 MHz in DMSO-d_6_ using tetramethylsilane as internal reference. Fluorescence intensity measurements were carried out on a Hitachi spectrofluorimeter model MPF-4. Melting point was measured on a Unimelt capillary melting point apparatus and reported uncorrected. Conductivity measurements of the above platinum and palladium complexes were carried out on a Systronics conductivity bridge 305, using a conductivity cell of cell constant 1.0. Doubly distilled water was used as solvent all along. 

### 2.2. Synthesis of Octyldithiocarbamate Sodium Salt (Oct-dtcNa)

**Scheme 1:**
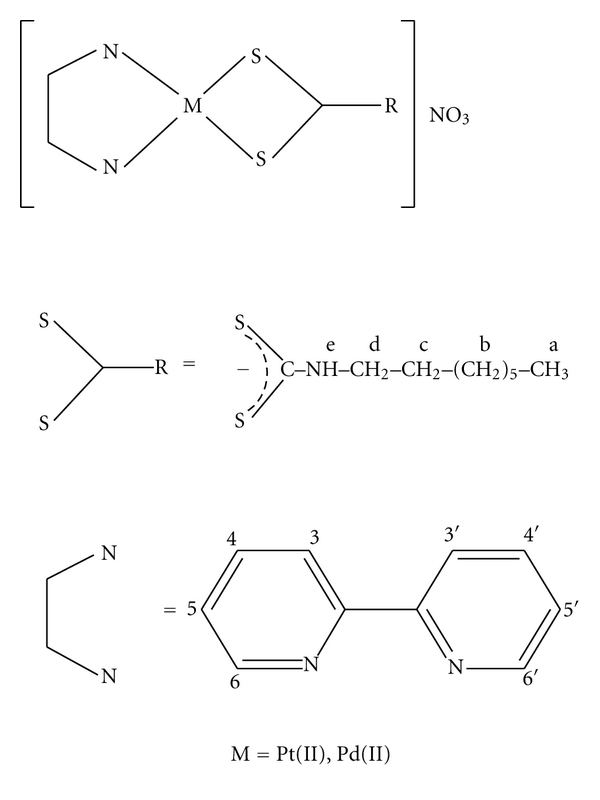
Proposed structures and proton NMR numbering schemes of [Pt/Pd(bpy)(Oct-dtc)]NO_3_ complexes.

This ligand was synthesized by the method as described earlier [15], except that butylamine was replaced by octylamine (8.33 mL, 50 mmol). The yield was 8.51 g (75%); m.p. 173°C. Anal. Calcd. For C_9_H_18_NS_2_Na (227 g/mol): C, 47.58; H, 7.93; N, 6.17%. Found: C, 47.64; H, 7.92; N, 6.16%. Solid state IR spectroscopy of the above ligand shows three main characteristic bands at 1491, 930, and 3171 cm^−1^ assigned to *ν* (N-CSS) and *ν* (SCS) and *ν* (N-H) stretching modes, respectively, [16]. ^1^H NMR (500 MHz, DMSO-d_6_, ppm, m = multiplet and sb = singlet broad): 0.84 (m, 3H, H-a), 1.24 (m, 10H, H-b), 1.41 (m, 2H, H-c), 3.31 (m, 2H, H-d), and 7.98 (sb, -NH-) (Scheme 1).

### 2.3. Synthesis of [Pt (Oct-dtc)(bpy)]NO_3_

This complex was synthesized following our previous procedure [15], except that But-dtcNa was replaced by Oct-dtcNa. The yield was 0.475 g, 77% and the complex decomposes at 197°C. Analysis calculated for C_19_H_26_  N_4_O_3_S_2_Pt (617): C, 36.95; H, 4.21; N, 9.08%. found: C, 36.90; H, 4.19; N, 9.07%. solid state FT-IR spectroscopy of the above complex shows three characteristic stretching bands at 1035, 1535 and 2945 cm^−1^ assigned to *ν*(SCS), *ν*(N-CSS) and *ν*(N-H) modes, respectively [20, 38]. The sharp band at 1385 cm^−1^ is assigned to uncoordinated NO_3_^−^ ion [39]. ^1^H NMR (500 MHz, DMSO-d_6_, ppm, Sb = singlet broad, t = triplet, d = doublet, q = quartet and m = multiplet [40]: 0.85 (t, 3H, H-a), 1.27 (m, 10H, H-b), 1.62 (t, 2H, H-c), 3.49 (t, 2H, H-d), 7.75 (t, 2H, H-4,4′), 8.45 (t, 2H, H-5,5′), 8.52 (q, 2H, H-3,3′), 8.70 (d, 2H, H-6,6′), and 11.53 (sb, 1H, H-e), (Scheme 1). Molar conductance of the complex is 91.78 *Ω* ^−1^ mol^−1^ cm^2^ indicating 1 : 1 electrolytes [41]. Electronic spectra exhibit five bands. The band at 364 (*ε* = 3.56) is assigned to MLCT and the other bands at 321 (*ε* = 6.72), 310 (*ε* = 5.42), 285 (*ε* = 13.69) and at 202 (*ε* = 27.41) may be due to first, second, and higher internal *π*-π* transitions of 2,2′-bipyridine as well as dithiocarbamate ligands [15].

### 2.4. Synthesis of [Pd(Oct-dtc)(bpy)]NO_3_

This complex was prepared by a similar method to that of [Pt(bpy)(Oct-dtc)]NO_3_. Yield was 0.360 g, 68% and decomposes at 178°C. Analysis calculated for C_19_H_26_N_4_O_3_S_2_Pd (528): C, 43.18; H, 4.92; N, 10.61%. Found: C, 43.17; H, 4.93; N, 10.60%. Solid state FT-IR spectroscopy of the complex shows three characteristic stretching bands at 1539, 1030, and 2987 cm^−1^ assigned to *ν*(N-CSS), *ν*(SCS), and *ν*(N-H) modes, respectively, [20, 38]. The sharp band at 1385 cm^−1^ is assigned to uncoordinated NO_3_^−^ ion [39]. ^1^H NMR (500 MHz, DMSO-d_6_, ppm, sb = singlet broad, t = triplet, q = quartet, and m = multiplet (40): 0.82 (t, 3H, H-a), 1.24 (m, 10H, H-b), 1.57 (m, 2H, H-c), 3.45 (t, 2H, H-d), 7.69 (m, 2H, H-4,4′), 8.15 (q, 2H, H-5,5′), 8.26 (m, 2H, H-3,3′), 8.51 (q, 2H, H-6,6′), and 11.21 (sb, 1H, H-e) (Scheme 1). Molar conductance of the complex is 97 Ω^−1 ^mol^−1^ cm^2^ indicating 1 : 1 electrolytes [41]. Electronic spectra exhibit four bands. The bands at 318 (*ε* = 1.88) and 307 (*ε* = 1.82) are assigned to MLCT, and the other bands at 247 (*ε* = 6.50) and 203 (*ε* = 3.78) may be assigned to first and second intraligand *π*-π* transition of 2,2′-bipyridine ligand as well as −CSS^−^ group of dithiocarbamate ligand [15].

### 2.5. Cytotoxic Studies

The procedure for cytotoxic studies of the [Pt(bpy)(Oct-dtc)]NO_3_ and [Pd(bpy)(Oct-dtc)]NO_3_ was similar to that reported earlier [15]. Here, also 1 × 10^4^ cells per mL of K562 chronic myelogenous leukemia were used in Tris-HCl buffer solution of PH 7.0.

### 2.6. Metal Complexes—DNA-Binding Studies

[Pt/Pd(bpy)(Oct-dtc)]NO_3_ complexes were interacted with calf thymus DNA in Tris-HCl buffer (20 mM, PH = 7.0) containing 20 mM sodium chloride. The procedure followed to determine binding and thermodynamic parameters were similar to what was reported earlier [16]. The stock solutions of the complexes (0.5 mmol/L) and CT-DNA (4 mg/mL) were made in the same buffer. The DNA-metal complex solutions were incubated at 300 K and 310 K, and, then, the spectrophotometric reading at *λ*_max⁡_ of the complexes (321 nm for Pt(II) complex and 305 nm for pd(II) complex), where CT-DNA has no absorption, were measured. Using trial and error method, the incubation time for solutions of DNA-metal complexes at 300 K and 310 K was found to be 6 h. No further changes were observed in the absorbance reading after longer incubation. All the experiments repeated to get the constant results.

## 3. Results and Discussion

The compounds correspond to the composition Oct-dtcNa, [Pd(bpy)(Oct-dtc)]NO_3_ and [Pt(bpy)(Oct-dtc)]NO_3_, where bpy = 2,2′-bipyridine, and Oct-dtc = octyldithiocarbamato ligands were prepared and characterized by chemical analysis, conductance measurements, ultraviolet-visible, infrared and, ^1^H NMR spectroscopic methods. These analytical data of the complexes are given in experimental section and the proposed structure in Scheme 1. Cytotoxicity and detailed calf thymus DNA-binding studies of these water-soluble complexes have been studied. 

### 3.1. Cytotoxic Measurement of the Metal Complexes

**Figure 1:**
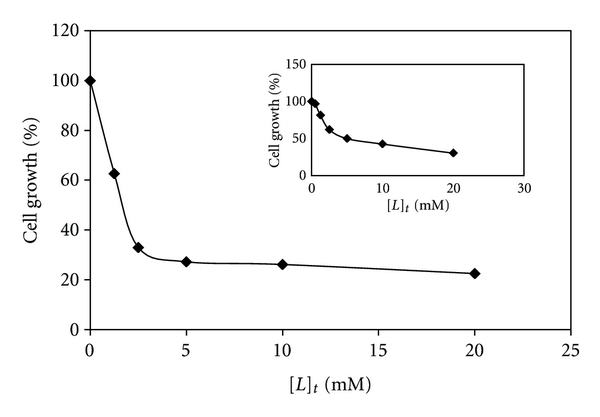
The growth suppression activity of the Pt(II) complex and the inset for Pd(II) complex on K562 cell line assessed using MTT assay as described in Section 2.1. The tumor cells were incubated with varying concentrations of the complexes for 24 h.

[Pt/Pd(bpy)(Oct-dtc)]NO_3_ complexes were screened for their anti-tumor activities against K562 chronic myelogenous leukemia cells [15]. These cells were maintained in RPMI 1640 medium supplemented with 10% FCS in a humidified incubator (310 K and 5% CO_2_). The cells were then grown in RPMI medium supplemented with *L*-glutamine (2 mM), streptomycin and penicillin (5 *μ*g/mL), and 10% heat-inactivated fetal calf serum, at 310 K under a 5% CO_2_/95% air atmosphere. In this study, the harvested cells were seeded into 96-well plate (1 × 10^4^ cell/mL) with various concentrations of metal complexes ranging from 0 to 0.25 mM and incubated for 24 h [42]. The 50% cytotoxic concentrations (Ic_50_) of the Pt(II) and Pd(II) complexes were determined to be 0.0017 and 0.007 mM, respectively (Figure 1 for pt(II) complex and the inset for Pd(II) complex). As shown in Figure 1, cell growing after 24 h was significantly reduced in the presence of various concentrations of the metal complexes. Furthermore, Ic_50_ value of cisplatin under the same experimental conditions was determined. This value (0.154 mM) is much higher as compared to the Ic_50_ value of the above two complexes. However, the Ic_50_ values of these complexes are comparable with those of our analogous Pt(II) and Pd(II) dithiocarbamate complexes reported earlier [15–17]. This procedure for growth inhibition studies of the metal complexes established that the cell DNA is the target biomolecule for these complexes [39].

### 3.2. DNA-Binding Studies

#### 3.2.1. CT-DNA Denaturation Data and Evaluation of Thermodynamic Parameters

Absorption spectroscopy is one of the most useful techniques to study the binding of any drug to DNA [43–46]. The procedure followed was similar to that reported earlier [47, 48]. These experiments were carried out separately at two temperatures of 300 K and 310 K in Tris-HCl buffer medium. The absorbance at 260 nm was monitored for either CT-DNA (0.129 mM and 0.115 mM for experiments carried out for Pt(II) complex and 0.197 mM and 0.187 mM for Pd(II) complex at 300 K and 310 K, resp.) or mixtures of CT-DNA with increasing concentrations of Pt(II) and Pd(II) complexes (0.0028 mM to 0.00130 mM and 0.0028 mM to 0.114 mM for Pt(II) complex and 0.0068 mM to 0.140 mM and 0.0028 mM to 0.113 mM for Pd(II) complex at 300 K and 310 K, resp.). Also the absorbance of CT-DNA and mixture of DNA-Pt(II)/Pd(II) complexes was measured at 640 nm to eliminate the interference of turbidity. 

**Figure 2:**
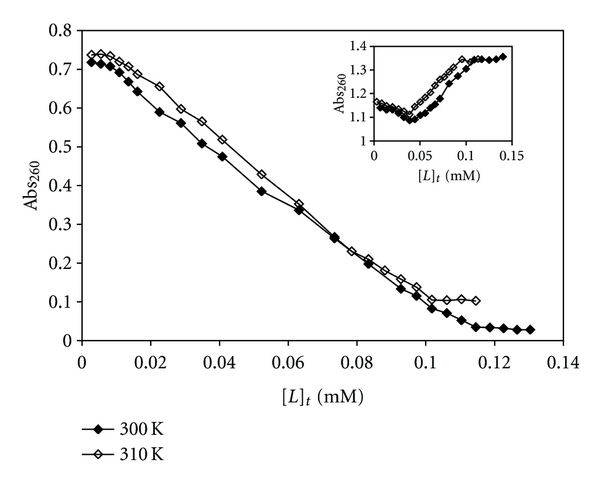
The changes of absorbance of CT-DNA at *λ*_max⁡_ = 260 nm due to increasing the total concentration of [Pt(bpy)(Oct-dtc)]NO_3_ and the inset for [Pd(bpy)(Oct-dtc)]NO_3_, [*L*]_t_, at constant temperatures of 300 K and 310 K.

The profiles of denaturation of CT-DNA by [Pt(Oct-dtc)(bpy)]NO_3_ and [Pd(Oct-dtc)(bpy)]NO_3_ are shown in Figure 2. As Figure 2 shows, the concentration of metal complexes in the midpoint of transition, [*L*]_1/2_, at 300 K is 0.065 mM and at 310 K is 0.059 mM for Pt(II) complex and 0.093 mM at 300 K and 0.086 mM at 310 K for Pd(II) complex. One of the most important observations made in this research is the extraordinary low values of [L]_1/2_ for [Pt(Oct-dtc)(bpy)]NO_3_ and [Pd(Oct-dtc)(bpy)]NO_3_ complexes. This means that the complexes can denature CT-DNA at extremely low concentrations, and if these will be used as anticancer agent, quite low doses will be needed, which may have fewer side effects. These values are lower or comparable with [*L*]_1/2_ values of binding of analogous complexes [Pt/Pd(bpy)(Et-dtc)]NO_3_ [15] and [Pt/Pd(bpy)(Bu-dtc)]NO_3_ [16] with CT-DNA. 

It is noticeable that absorbance of DNA at 260 nm should increase in presence of increasing amount of each metal complex (denaturant agent). This is true for palladium complex (Figure 2). However, the opposite trend is observed for the platinum analog. Keeping in mind that platinum complexes react about 10^5^–10^6^ times slower than palladium complexes [49, 50], the decrease in the absorbance at 260 nm with the increase of the amount of Pt(II) complex added to CT-DNA may be due to (i) a possibility that interaction between CT-DNA and the metal complex causes the double helix of CT-DNA to become more straight leading to stacking; this stacking may cause conformational changes leading to a sort of denaturation or (ii) each strand after denaturation gets associated in a more stacked structure and (iii) metal complex slips into the helix and masks the hydrophobic bases leading to a decrease in absorbance. As will be seen in the later part of this paper, the [Pt(Oct-dtc)(bpy)]NO_3_ and [Pd(Oct-dtc)(bpy)]NO_3_ complexes can bind CT-DNA taking the mode of intercalation. This mode of binding supports the above three hypotheses. 

**Figure 3:**
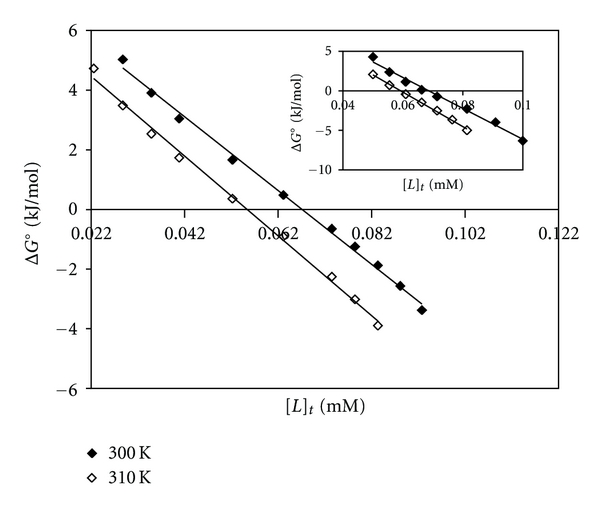
The molar Gibbs free energies of unfolding (Δ*G*° versus [*L*]_t_) of CT-DNA in the presence of [Pt(bpy)(Oct-dtc)]NO_3_ and the inset for [Pd(bpy)(Oct-dtc)]NO_3_.

Using the CT-DNA denaturation plots (Figure 2) and Pace method [50], the value of *K*, that is, unfolding equilibrium constant and Δ*G*°, unfolding free energy of CT-DNA, at two temperatures of 300 K and at 310 K in the presence of Pt(II) and Pd(II) complexes have been calculated. In this method, Pace had assumed two-state mechanism, nature and denature, and then calculated unfolding free energy of DNA, that is, (ΔG°) by using (1):

(1) $$\begin{array}{c} K=\frac{A_{N}-A_{\text{obs}}}{A_{0\text{bs}}-A_{D}},\\ \Delta G_{}^{{}^{\circ}}=-\text{RT}\;Ln\;K, \end{array}$$

where *A*_obs_ is absorbance readings in transition region and *A*_N_ and *A*_D_ are absorbance readings of nature and denatured conformation of DNA, respectively. A straight line is observed when the values of Δ*G*° are plotted against the concentrations of each metal complex in the transition region at 300 K and at 310 K. These plots are shown in Figure 3 for Pt(II) complex and the inset for Pd(II) complex. The equation for these lines can be written as follow [51]:

(2) $$\;\Delta G^{{}^{\circ}}={\Delta G^{{}^{\circ}}}_{\left({\text{H}}_{2}\text{O}\right)}-m\left[\text{complex}\right].$$

**Figure 4:**
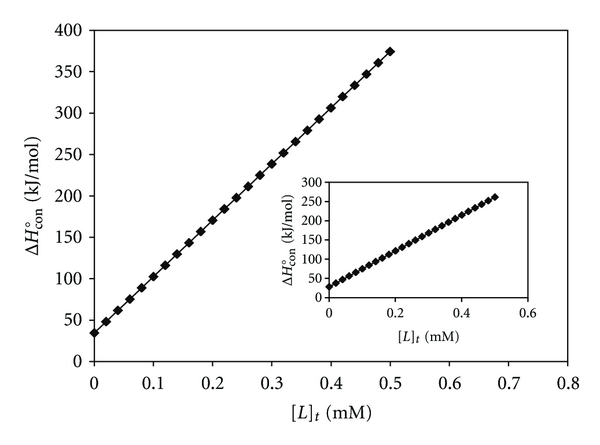
The molar enthalpies of CT-DNA denaturation in the interaction with [Pt(bpy)(Oct-dtc)]NO_3_ and the inset for [Pd(bpy)(Oct-dtc)]NO_3_ complexes in the range of 300 K to 310 K.

Here, the values of Δ*G*_(H_2_O)_° for each curve are measured from the intercept on ordinate of the plots and it is conformational stability of DNA in the absence of metal complex. *m* (the slope of each curve in the same plots) is a measure of the metal complex ability to denature CT-DNA and is summarized in Table 1. The values of  *m*  for the above complexes are much higher than those of Pt(II) and Pd(II) complexes reported earlier [15–17], which indicate the higher ability of this Pt(II) and Pd(II) complexes to denature CT-DNA. As we know, the higher the values of Δ*G*°, the larger the conformational stability of CT-DNA. However, the values of Δ*G*° (Table 1) are decreased by increasing the temperature. This is as expected because, in general, the decrease in Δ*G*_(H_2_O)_° value is the main reason for the decrease in CT-DNA stability [52]. Molar enthalpy of CT-DNA denaturation in absence of Pt(II) and Pd(II) complexes, Δ*H*_(H_2_O)_°, is another important thermodynamic parameter. To find this, we calculated the molar enthalpy of CT-DNA denaturation in presence of the metal complexes, Δ*H*°_conformation_ or Δ*H*°_denaturation_, in the range of two temperatures using Gibbs-Helmholtz equation [53]. On plotting the values of these enthalpies versus the concentration of metal complexes, straight lines will be obtained which are shown in Figure 4 for [Pt(Oct-dtc)(bpy)]NO_3_ and the inset for [Pd(Oct-dtc)(bpy)]NO_3_ complexes. Intrapolation of these lines (intercept on ordinate, i.e., absence of metal complexes) gives the values of Δ*H*_(H_2_O)_° (Table 1). These plots show that in the range of 300–310 K the changes in the enthalpies in presence of Pt(II) and Pd(II) complexes are ascending. These observations indicate that, on increasing the concentration of Pt(II) and Pd(II) complexes, the stability of CT-DNA is increased. Moreover, the entropy of CT-DNA unfolding by Pt(II) and Pd(II) complexes Δ*S*_(H_2_O)_° has been calculated using equation Δ*G* = Δ*H* − *T*Δ*S* and the data are given in Table 1. These data show that the metal-DNA complexes are more disordered than those of native CT-DNA, because the entropy changes are positive for Pt(II)- or Pd(II)-DNA complexes in the denaturation processes of CT-DNA (Table 1). The above thermodynamic parameters agree well with those we have reported for [Pt/Pd(bpy)(Et-dtc)]NO_3_ [15] and [Pt/Pd(bpy)(Bu-dtc)]NO_3_ [16] complexes. 

#### 3.2.2. UV-Vis Spectral Studies and Evaluation of Binding Parameters

**Figure 5:**
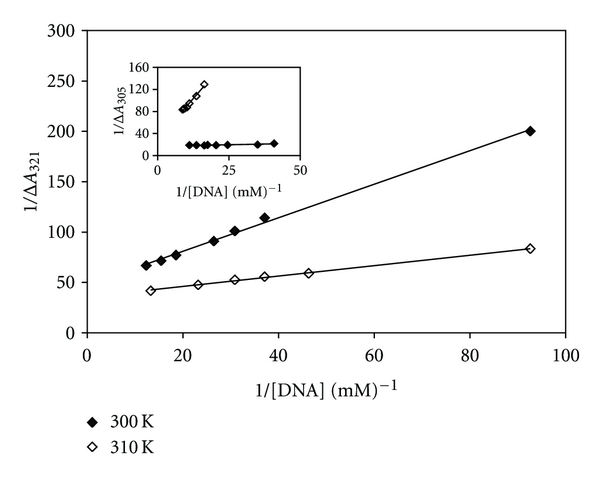
The changes in the absorbance of fixed amount of each metal complex in the interaction with varying amount of CT-DNA at 300 K and 310 K. The linear plot of the reciprocal of Δ*A* versus the reciprocal of [DNA] for [Pt(bpy)(Oct-dtc)]NO_3_ and the inset for [Pd(bpy)(Oct-dtc)]NO_3_.

**Figure 6:**
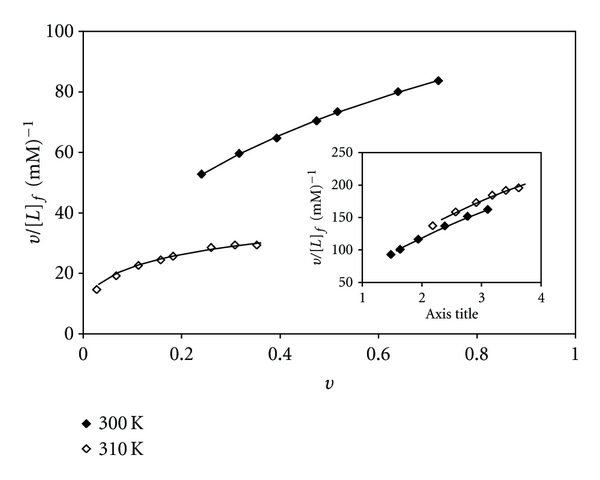
Scatchard plots for binding of [Pt(bpy)(Oct-dtc)]NO_3_ and the inset for [Pd(bpy)(Oct-dtc)]NO_3_ to CT-DNA.

A fixed amount of [Pt(Oct-dtc)(bpy)]NO_3_ and [Pd(Oct-dtc)(bpy)]NO_3_ complexes (0.006 mM for Pt(II) complex and 0.025 mM and 0.037 mM for Pd(II) complex at 300 K and 310 K, resp.) was titrated with increasing amounts of CT-DNA (0.010 mM to 0.081 mM and 0.010 mM to 0.076 mM for Pt(II) complex and 0.024 mM to 0.089 mM and 0.061 to 0.114 mM for Pd(II) complex at 300 K and 310 K, resp.). In this experiment, the changes in the absorbance, Δ*A*, of metal complexes at 321 nm for Pt(II) complex and 305 nm for Pd(II) complex were calculated by subtracting the absorbance reading of DNA-metal complexes solution from absorbance reading of free metal complexes solution. The maximum Δ*A* (Δ*A*_max⁡_), that is, change in the absorbance when all binding sites on CT-DNA were occupied by metal complex (Figure 5, intercept on ordinate) is 0.021 and 0.028 for Pt(II) complex and 0.035 and 0.058 for Pd(II) complex at 300 K and 310 K, respectively. These values of Δ*A*_max⁡_ were used to calculate the concentration of bound metal complexes to CT-DNA in the next experiment: a fixed amount of CT-DNA (0.023 mM for Pt(II) complex and 0.001 mM for Pd(II) complex) was titrated with varying concentrations of Pt(II) and Pd(II) complexes (10 to 0.025 mM and 0.002 to 0.020 mM for Pt(II) complex and 0.017 to 0.022 mM and 0.018 to 0.022 mM for Pd(II) complex at 300 K and 310 K, resp.). Now, the concentrations of metal complexes bound to CT-DNA, [*L*]_b_, and the concentration of free metal complex, [*L*]_f_, are calculated using the relationship

(3) $$\begin{array}{c} {\left[L\right]}_{b}=\Delta A\;{\left[L\right]}_{t}/\Delta A_{\max},\\ {\left[L\right]}_{f}={\left[L\right]}_{t}-{\left[L\right]}_{b}, \end{array}$$

where [*L*]_t_ is the maximum concentration of metal complex added to saturate all the binding sites of CT-DNA. The Scatchard plots were obtained separately at 300 K and 310 K by plotting *ν*/[*L*]_t_ versus *ν* of the relationship *ν* = [*L*]_b_/[DNA]_t_ (Figure 6). These plots are curvilinear concave downwards, suggesting cooperative binding [54]. Similar cooperativity in binding of analogous complexes with CT-DNA has also been observed [15, 55]. 

On substituting the above experimental data (*ν* and [*L*]_f_) in Hill equation,

(4) $$\nu =\frac{g{\left(K{\left[L\right]}_{f}\right)}^{n}}{{\left(1+\text{(}K\;{\left[L\right]}_{f}\right)}^{n}},$$

we get a series of equations with unknown binding parameters *n*, *K*, and *g*. Using Eureka software, the theoretical values of these parameters have been deduced. The results are shown in Table 2. These results are comparable with those of 2,2′-bipyridine-platinum and -palladium complexes of dithiocarbamates as reported earlier [15, 16]. The apparent binding constants of two complexes obtained were 4.01 × 10^4^ M^−1^ at 300 K and 1.10 × 10^4^ M^−1^ at 310 K for Pt(II) complex and 5.28 × 10^4^ M^−1^ at 300 K and 5.86 × 10^4^ M^−1^ at 310 K for Pd(II) complex. The values are comparable to those of CT-DNA intercalators [Pd(dmphen)CO_3_]·H_2_O 1.6 × 10^4^ M^−1^ [56] and [Cu(GFL)(A^1^)Cl]·5H_2_O 2.45 × 10^3^ M^−1^ [57] and [Ru(bpy)_2_(PPIP)]_2_^2+^ *‏* 4.3 × 10^4^ M^−1^ [58] and [Ru (dmb)_2_(NMIP)]_2*‏*_ 5.46 × 10^3^ M^−1^ [59]. Obviously, these thermodynamical characteristics suggest that the two complexes intercalatively bind to CT-DNA, involving a strong stacking interaction between the aromatic chromophore and the base pairs of the DNA [60]. Similar results were obtained for [Pd(ddtc) (bpy)]NO_3_·H_2_O [7]. The maximum errors between experimental and theoretical values of *ν* are also shown in Table 2, which are quite low.

**Figure 7:**
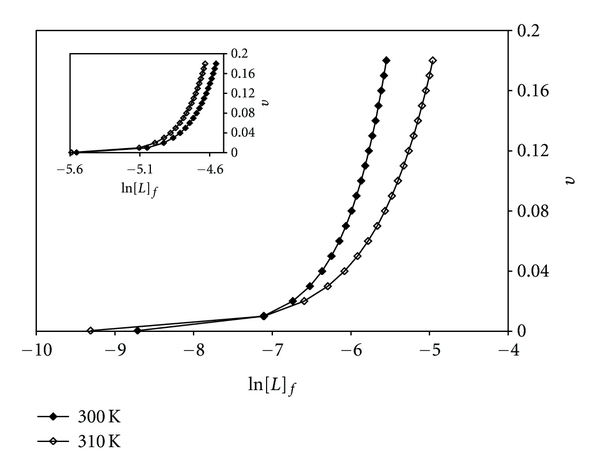
Binding isotherm plots for [Pt(bpy)(Oct-dtc)]NO_3_ and the inset for [Pd(bpy)(Oct-dtc)]NO_3_ in the interaction with CT-DNA.

**Figure 8:**
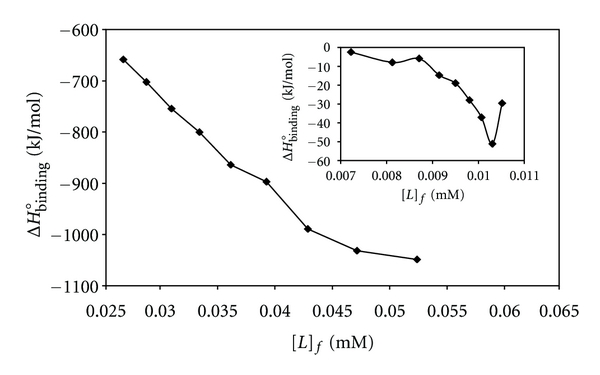
Molar enthalpies of binding in the interaction between CT-DNA and [Pt(bpy)(Oct-dtc)]NO_3_ and the inset for [Pd(bpy)(Oct-dtc)]NO_3_ versus free concentrations of complexes at pH 7.0 and 300 K.

 Knowing the experimental (dots) and theoretical (lines) values of *ν* in the Scatchard plots and their superimposability on each other, these values of *ν* were plotted versus the values of ln[*L*]_f_. The results are sigmoidal curves and are shown in Figure 7 for Pt(II) complex and the inset for Pd(II) complex at 300 K and 310 K. These plots indicate positive cooperative binding at both temperatures for the complexes. The area under these plots of binding isotherms were found and by using Wyman-Jons equation [61], *K*_app_, Δ*G*°_b_ and Δ*H*°_b_ were calculated at 300 K and 310 K for each particular *ν*. Plots of the values of Δ*H*°_b_ versus the values of [*L*]_f_ are shown in Figure 8 for [Pt(Oct-dtc)(bpy)]NO_3_ and the inset for [Pd(Oct-dtc)(bpy)]NO_3_ at 300 K. These plots show that, at very low value of [*L*]_f_ (~0.05 mM for Pt(II) complex and ~0.01 mM for Pd(II) complex), available binding sites for the metal complexes on CT-DNA have been saturated. This may be due to high interaction affinity of CT-DNA for [Pt(Oct-dtc)(bpy)]NO_3_ and [Pd(Oct-dtc)(bpy)]NO_3_ complexes [61, 62]. 

### 3.3. Gel Filtration

**Figure 9:**
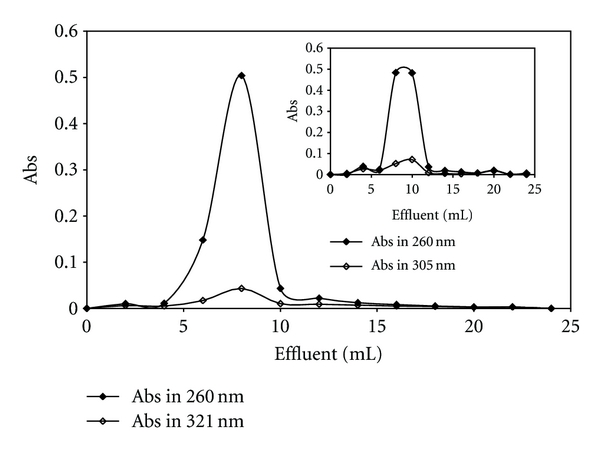
Gel chromatograms of [Pt(bpy)(Oct-dtc)]NO_3_ and the inset for [Pd(bpy)(Oct-dtc)]NO_3_ obtained on Sephadex G-25 column.

 [Pt(Oct-dtc)(bpy)]NO_3_ and [Pd(Oct-dtc)(bpy)]NO_3_ complexes (0.125 mM and 0.050 mM, resp.) were incubated with CT-DNA (0.223 mM for Pt(II) complex and 0.249 mM for Pd(II) complex) for 6 h at 300 K in Tris-HCl buffer, pH 7.0. DNA-metal complexes were then passed through a Sephadex G-25 column equilibrated with the same buffer. The elusion of the column fraction of 2.0 mL was monitored at 321 nm and 260 nm for DNA-Pt(II) complex system and at 305 nm and 260 nm for DNA-Pd(II) complex system. These results are given in Figure 9 for Pt(II) complex and the inset for Pd(II) complex. This plot shows that the peak obtained for the two wavelengths are not resolved and suggests that CT-DNA has not separated from the metal complexes. Thus, it implies that the binding between CT-DNA and the metal complexes is not reversible under such circumstances. This is due to the fact that if the interaction between CT-DNA and metal complexes was weak, the CT-DNA should have come out of the column separately [63]. 

## 4. Conclusions

The above work describes the synthesis, characterization, and cytotoxic studies of a novel and water-soluble platinum(II) and palladium(II) complexes possessing a planar aromatic ligand for intercalation and a dithiocarbamate ligand to avoid its renal damage. Ic_50_ values of these complexes are much lower than those of cisplatin. Furthermore, detailed interaction studies of the complexes with CT-DNA have been carried out. They cooperatively bind to DNA through intercalation mode and unexpectedly denature DNA at extremely low concentration. Determinations of several binding and thermodynamic parameters have also been attempted. They may be helpful to understand the mechanism of the interaction between these complex and nucleic acid that must be quite different from that of cisplatin. 

### 4.1. Competitive Binding between Ethidium Bromide (EB) and Pt(II)/Pd(II) Complexes for CT-DNA

**Figure 10:**
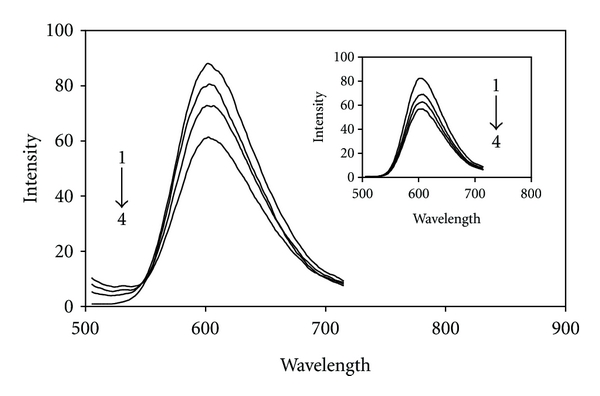
Fluorescence emission spectra of interacted EB-DNA in the absence (1) and presence of different concentrations of [Pt(bpy)(Oct-dtc)]NO_3_ and the inset for [Pd(bpy)(Oct-dtc)]NO_3_: 33  *μ*M (2), 71 *μΜ* (3), and 111 *μ*M (4) and 12 *μ*M (2), 35 *μ*M (3), and 55 *μ*M (4), respectively, at 300 K.

No fluorescence was observed for aqueous solution of above Pt(II) and Pd(II) complexes alone or in the presence of calf thymus DNA. So, the binding of Pt(II) and Pd(II) complexes with CT-DNA cannot be directly presented in the emission spectra. It has been studied by competitive EB binding experiments [64, 65]. The fluorescence of EB is greatly enhanced upon intercalation to DNA. The effect of platinum(II)-2,2′-bipyridine and palladium(II)-2,2′-bipyridine complexes of octyldithiocarbamate on the fluorescence intensity of DNA-EB complexes was studied to get the mode of their binding to CT-DNA.  Figure 10 shows the effect of Pt(II) and Pd(II) complexes (33 *μ*M, 71 *μ*M and 111 *μ*M for Pt(II) complex and 12 *μ*M, 35 *μ*M and 55 *μ*M for Pd(II) complex) on fluorescence spectrum of solution containing CT-DNA (60 *μ*M) and EB (2 *μ*M). It is seen that increasing the concentration of the Pt(II)/Pd(II) complexes results in a gradual decrease in fluorescence intensity of DNA-EB solution, without effecting any perceptible shifts in fluorescence *λ*_max⁡_. It further proves the interaction between these complexes and DNA molecules. A similar fluorescence quenching behavior was observed for analogous Pt(II)/Pd(II) complexes reported earlier [47, 48]. 

**Figure 11:**
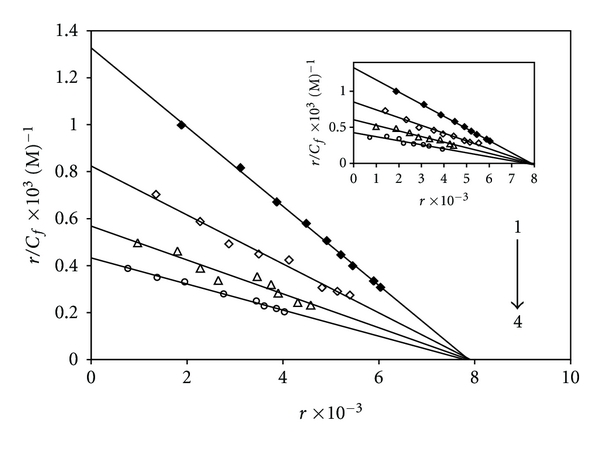
Competition between [Pt(bpy)(Oct-dtc)]NO_3_ and the inset for [Pd(bpy)(Oct-dtc)]NO_3_ with ethidium bromide for the binding sites of DNA (Scatchard plot). In curve no. 1, Scatchard plot was obtained with calf thymus DNA alone. Its concentration was 60 *μ*M. In curves nos. 2, 3, and 4, respectively, 33 *μ*M, 71 *μ*M, and 111 *μ*M for Pt(II) complex and 12 *μ*M, 35 *μ*M, and 55 *μ*M, for Pd(II) complex were added, corresponding to molar ratio [complex]/[DNA] of 0.55, 1.18, and 1.85 for Pt(II) complex and 0.2, 0.58, and 0.92 for Pd(II) complex. Solutions were in 20 mM NaCl and 20 mM Tris-HCl (pH 7.0). Experiments were done at room temperature.

Further studies to characterize the mode of binding of [Pt(Oct-dtc)(bpy)]NO_3_ and [Pd(Oct-dtc)(bpy)]NO_3_ to DNA were carried out [66, 67]. The number of EB molecules intercalated to CT-DNA in presence of different concentrations of the Pt(II) complex and Pd(II) complex was calculated using Scatchard analysis [68]. In this experiment, the wavelengths of excitation and emission were set at 540 nm and 700 nm, respectively, with both having 0.5 nm slit widths. Solutions of CT-DNA, EB, and metal complexes were prepared in Tris-HCl buffer of pH 7.0. In this medium-solutions of Pt(II) complex and Pd(II) complex were interacted with CT-DNA by incubating them at 300 K and 310 K for 6 h; appropriate amount of EB was added to them and further incubated at room temperature (300 K) for 6 h and finally processed for fluorescence spectral measurement. Saturation curves of fluorescence intensity for [Pt/Pd(Oct-dtc)(bpy)]^+^-DNA systems at different *r*_*f*_ values (0.55, 1.18, and 1.85 for Pt(II) complex and 0.2, 0.58 and 0.92 for Pd(II) complex) in presence of increasing concentrations of EB (2, 4,…, 20 *μ*M) were obtained. The fluorescence Scatchard plots obtained for binding of EB to CT-DNA in absence (♦) and presence (*◊*, Δ, and ∘) of various concentration of Pt(II) and Pd(II) (inset) complexes were shown in Figure 11. This figure shows that the complexes inhibit competitively the EB binding to CT-DNA (type A behavior) [68], where number of binding sites *n* (intercept on the abscissa) remains constant and the slope of the graphs that is *K*_app_ (apparent association constant) decreases with increasing the concentrations of Pt(II) and Pd(II) complexes (Table 3). This implies that the [Pt/Pd(Oct-dtc)(bpy)]NO_3_ complexes are intercalating in CT-DNA and thereby competing for intercalation sites occupied by EB. The values of *K*_app_ and *n*, the number of binding sites per nucleotide, are listed in Table 3. These data suggested that the interaction of the [Pd(Oct-dtc)(bpy)]NO_3_ complex with CT-DNA was stronger than that of [Pt(Oct-dtc)(bpy)]NO_3_ complex, which were consistent with the above absorption spectral results. Compare their *K*_app_ values with those of other known CT-DNA-intercalative complexes which possess analogical structure; the Pd(II) complexes in our paper have similar or stronger affinities with CT-DNA [17]. Similar modes of binding seem to be involved in other complexes [52, 69]. 

## Figures and Tables

**Table 1:** Thermodynamic parameters of CT-DNA denaturation by palladium(II) and platinum(II) complexes.

Compound	Temperature (*K*)	^ a^ *m* (kJ/mol) (mmol/L)^−1^	^ b^Δ*G*_(H_2_O)_° (kJ/molK)	^ c^Δ*H*_(H_2_O)_° (kJ/mol)	^ d^Δ*S*_(H_2_O)_° (kJ/mol)
[Pt(bpy)(Oct-dtc)]NO_3_	300	123	8.30	34.5	0.08
	310	133	7.40		
[Pd(bpy)(Oct-dtc)]NO_3_	300	195	13.44	28.09	0.05
	310	218	12.93		

^a^Measure of the metal complex ability to denature CT-DNA.

^b^Conformational stability of CT-DNA in the absence of metal complex.

^c^The heat needed for CT-DNA denaturation in the absence of metal complex.

^d^The entropy of CT-DNA denaturation by metal complex.

**Table 2:** Values of Δ*A*_max _ and binding parameters in the Hill equation for interaction between Pt(II) and Pd(II) complexes and CT-DNA in 20 mmol/L Tris-HCl buffer and pH 7.0.

Compound	Temperature (*K*)	^ a^Δ*A*_max _	^ b^ *g*	^ c^ *K* × 10^4^ (M)^−1^	^ d^ *n*	^ e^Error
[Pt(bpy)(Oct-dtc)]NO_3_	300	0.021	6	4.01	1.87	0.005
	310	0.028		1.10	1.36	0.007
[Pd(bpy)(Oct-dtc)]NO_3_	300	0.035	6	5.28	5.92	0.13
	310	0.058		5.86	6.18	0.14

^a^Change in the absorbance when all the binding sites on CT-DNA were occupied by metal complex.

^b^The number of binding sites per 1000 nucleotides.

^c^The apparent binding constant.

^d^The Hill coefficient (as a criterion of cooperativity).

^e^Maximum error between theoretical and experimental values of *ν*.

**Table 3:** Binding parameters for the effect of platinum and palladium complexes on the fluorescence of EBr in the presence of CT-DNA.

Compound	^ a^ *r*_f_	^ b^ *K* × 10^5^ (M)^−1^	^ c^ *n*
[Pt/Pd(bpy)(Oct-dtc)]NO_3_	0.00	1.68	0.0078
[Pt(bpy)(Oct-dtc)]NO_3_	0.55	1.04	
	1.185	0.72	
	1.85	0.55	
[Pd(bpy)(Oct-dtc)]NO_3_	0.2	1.08	0.0078
	0.58	0.78	
	0.92	0.56	

^a^Formal ratio of metal complex to nucleotide concentration.

^b^Association constant.

^c^Number of binding sites (*n*) per nucleotide.
